# Reversible Long Term Contraceptives Utilization among Married Women of Reproductive Age Group in Areka Town, Southern Ethiopia

**DOI:** 10.4314/ejhs.v30i6.9

**Published:** 2020-11

**Authors:** Samson Kastro Dake, Temesgen Lera Abiso

**Affiliations:** 1 Department of Reproductive Health and Nutrition, Wolaita Sodo University, Sodo, Ethiopia

**Keywords:** Reversible long term contraceptives, Married women, reproductive age group, Areka Town, South Ethiopia

## Abstract

**Background:**

In low income countries, bearing many children is the main factor affecting maternal health. This study aimed to estimate the prevalence of reversible long term contraceptives utilization and identify factors associated with it among married women of child bearing age in Areka District in South Ethiopia

**Methods:**

We conducted a community-based cross-sectional survey involving systematically recruited 346 married women of reproductive age group. Data was collected using a structured interviewer-administered questionnaire on May 2019. We used SPSS version 25 for data entry and analyses. Bivariate logistic regression analysis was used to select exposure variables with crude association. Multivariate analysis was done to control for potential confounders and identify predictors of the outcome. Adjusted Odds Ratio (AOR) with 95% Confidence Interval (CI) was reported, and statistical significance was declared at p<0.05.

**Results:**

The prevalence of reversible long term contraceptives utilization among married women of reproductive age group was 134(38.7%). Utilization of Reversible Long Term Contraceptives (RLTCs) was positively associated with being protestant Christian religion follower, advanced educational status, history of abortion, and having a better attitude towards reversible long term contraceptives. In other words, being housewife, being daily laborer, having no radio in the household and making fertility decisions alone were negatively associated.

**Conclusion:**

The prevalence of RLTCs in the study area was high. Women should be empowered educationally through other alternative opportunities to formal school. In addition to electronic media, different community events and community conversations should be used to convey messages on contraceptives particularly RLTCs. Behavioral change communications would benefit women in shaping their attitudes towards RLTCs.

## Introduction

Maternal mortality remains high in most low-income countries. Africa is the region with the highest maternal mortality ratio and Ethiopia is one of the countries which contributed to this high maternal mortality ratio (401 deaths per 100,000 live births) (1). Bearing many children is among the factors which affect maternal health status. Ethiopia is the second most populous country in Africa with a fertility rate of 4.3 (2).

Globally, 190 million women want to avoid pregnancy and do not use any contraceptive method; only 19% of women rely on Reversible Long Term Contraceptives (RLTC). In sub-Saharan Africa, short acting contraceptive methods are most commonly used methods (3). Only 41% of currently married women use modern methods of family planning, and only 11% are using reversible long term contraceptives (4). Despite regional disparities in RLTCs utilization, the documented prevalence across Ethiopia ranges between 12.9% and 38% (5,6).

Evidences show that different factors hinder the utilization of RLTCs. Age, contraceptives knowledge, number of pregnancy, desire for more children, educational status, number of alive children, duration of family planning, spousal/partner support, ever use of RLTCs, fear of side-effects and source of contraception are some of the reported barriers.

Ethiopia, in the Health Sector Transformation Plan (HSTP V), planned to reduce maternal mortality from 420 to 199 by the end of the year 2020 (7). This cannot be achieved without increasing the prevalence of contraceptive utilization, particularly RLTCs. Since there are regional variations in the prevalence and factors associated with RLTCs, evidences are required for appropriate interventions. Thus, this study is aimed at determining the prevalence of RLTCs utilization and identifying factors associated with it.

## Methods

**Study setting**: Areka District, one of the districts in Wolaita Zone, South Ethiopia, is located at 329 km south from Addis Ababa. It is administratively structured into 4 kebeles (lower administrative structure), and it has a total population of 33,150 (8). One private hospital, one health center, and seven private clinics provide family planning and other health services to the population in the district.

**Study design and period**: A community-based cross-sectional study was conducted from May 1 to May 30, 2019.

**Population and sampling**: A total of 346 married women of child bearing age were systematically recruited. Women with known history of infertility or those who used non-reversible contraception were excluded from this study. A sample size of 352 was calculated using single population proportion formula with the following assumptions: 95% confidence level, 5% margin of error, estimated prevalence of 29.7% (9) and 10% non-response rate. Out of 4 kebeles, 2 kebeles were selected randomly, and the sample size was allocated to the selected kebeles proportionally. Households were selected systematically and if the selected household had more than one eligible woman, one was selected randomly.

**Measurements**: Eight questions were asked to assess women's knowledge about reversible long term contraceptives. The questions had five Likert scale responses: “Strongly disagree”, “Disagree”, “Not sure”, “Agree” and “Strongly agree”. A single variable was established by creating a total score from the questions. Women were categorized as having ‘good knowledge’ or ‘poor knowledge’ based on the mean score. Women with a score above the mean were labeled as having “better knowledge”, whereas those with a score of the mean or below were labeled as having “poor knowledge”. Cronbach's Alpha was used to check the reliability of the questions, and it was 0.81.

Similarly, to assess women's attitude towards reversible long term contraceptives, they were asked six Likert scale questions. Women with a score above the mean were categorized as having “better attitude”, whereas those with a score of the mean or below the mean were categorized as having “poor attitude”. Cronbach's Alpha was used to check the reliability of the questions, it was 0.79.

**Data collection**: Data were collected using a structured interviewer-administered questionnaire. The questionnaire was prepared after reviewing relevant articles and literature (10). The questionnaire was translated into the local language by fluent speakers of English and the local language. It was pretested with 18 respondents which were not included in the main study. Data collectors with prior experience of data collection were recruited and trained for two days on the data collection tool and ethical issues surrounding the study.

**Statistical analysis**: Data were entered, cleaned and analyzed by using SPSS version 25. Descriptive statistical techniques were used for all variables. Binary logistic regression analysis was used to select exposure variables with a crude association to the outcome variable. Hosmer-Lemeshow good-of-fit test was carried out to have confidence in the regression model. On bivariate analysis, a yardstick cut-off point of *p-value* < 0.25 was taken to select candidate variables for multivariate analysis. Multivariate analysis was done to control for potential confounders and identify predictors of the outcome. Adjusted Odds Ratio (AOR) with 95% Confidence Interval (CI) was reported, and statistical significance was declared at *p-value*< 0.05.

## Results

**Socio-demographic characteristics**: Three hundred and forty-six women were involved in this study with a response rate of 98.3%. Over three fourth (261,75.4%) were aged 20–35 years and 183(52.9%) were protestant Christian religion followers. Seventy-six (22.0%) women and 56(16.2%) husbands did not attend formal education. More than half (189, 54.6%) of the women were housewives whereas 150(43.4%) of husbands were governmental/non-governmental organizations (NGO) employees.

The majority (194,56.1%) of the households had an average monthly income of less than 1500 Ethiopian Birr (ETB). More than half (187,54.0%) of the households had radio and 246(71.1%) had television (Table 1).

**Table 1:** Socio-demographic characteristics of married women and their husbands at Areka town in South Ethiopia, May 2019

Variables (n=346)		Frequency	Percent
**Age (in years)**	<20	19	5.5
	20–35	261	75.4
	>35	66	19.1
**Religion**	Orthodox	97	28.0
	Muslim	35	10.1
	Protestant	183	52.9
	Others	31	9.0
**Educational status of**	No formal education	76	22.0
**women**	Primary school (1–8)	139	40.2
	Secondary and above	131	37.8
**Educational status of**	No formal education	56	16.2
**husbands**	Primary school (1–8)	81	23.4
	Secondary school (9–12)	82	23.7
	College and above	127	36.7
**Occupation of women**	Government/NGO employee	50	14.5
	Merchant	83	24.0
	Housewife	189	54.6
	Daily laborer	24	6.9
**Occupation of**	Government/NGO employee	150	43.4
**husbands**	Merchant	109	31.5
	Daily laborer	48	13.9
	Other	39	11.3
**Household income**	<1500	194	56.1
**(ETB)**	1500–3000	62	17.9
	>3000	90	26.0
**Have radio in the**	Yes	187	54.0
**household**	No	159	46.0
**Have television in the**	Yes	246	71.1
**household**	No	100	28.9

**Reproductive health characteristics, knowledge and attitude of women**: One fourth (88,25.4%) of the women gave birth to more than five children. Nearly three fourth (256,74.0%) had no history of abortion, and 202(58.4%) had a desire for future fertility. The majority (191, 55.2%) of the couples decide on future fertility jointly. More than half (182,52.6%) of the women had better knowledge whereas 154(44.5%) had better attitude about RLTC (Table 2).

**Table 2:** Reproductive characteristics, knowledge and attitude of women involved in the study at Areka town in South Ethiopia, May 2019

Variables (n=346)		Frequency	Percent
**Parity**	Nulliparous	14	4.0
	1–2	78	22.5
	3–5	166	48.0
	6+	88	25.4
**History of abortion**	Yes	90	26.0
	No	256	74.0
**Future fertility desire**	Yes	202	58.4
	No	144	41.6
**Decision maker on future**	Wife	55	15.9
**fertility**	Husband	100	28.9
	Jointly	191	55.2
**Knowledge about RLTC**	Good	182	52.6
	Poor	164	47.4
**Attitude on RLTC**	Good	154	44.5
	Poor	192	55.5

**Prevalence and predictors of RLTC utilization**: The prevalence of RLTC utilization among married women in this study was 134(38.7%); [95% CI= 33.6%–44.1%]. Protestant religion followers had about five times higher odds of using RLTC (AOR=5.33; 95% CI: 1.63, 17.40). Women who attended primary education were six times more likely to use RLTCs (AOR=5.78; 95% CI: 1.97, 17.02), whereas those who attended secondary education and above were seventeen times more likely to use RLTCs (AOR=16.69; 95% CI: 4.62, 60.31) compared to those with no formal education. The odds of utilizing RLTCs decreases by 73% for housewives (AOR=.27; 95% CI: .09, .79) and by 86% for daily laborers compared to government and NGO employees (AOR=.14; 95% CI: .02, .80).

The odds of using RLTCs decreases by 60% for women who do not have radio in the household (AOR=.40; 95% CI: .16, .99). Women who had history of abortion were seven times more likely to use RLTCs (AOR=7.17; 95% CI: 2.81, 18.27). On the other hand, women who decided alone on future fertility were 88% less likely to use RLTCs compared with those who decide jointly (AOR=.12; 95% CI: .04, .37). Women with a better attitude about RLTCs were three times more likely to utilize RLTCs (AOR=2.85; 95% CI: 1.46, 5.57) (Table 3).

**Table 3:** Factors associated with reversible long term contraceptive utilization in Areka town, South Ethiopia, May 2019

Variable (n=346)	RLTC utilization		COR (96% CI)	AOR (95% CI)
			
	Yes	No		
**Religion**				
Muslim	11(31.4)	24(68.8)	1	1
Orthodox	45(46.4)	52(53.6)	1.89(.83, 4.28)	1.02(.29, 3.65)
Protestant	69(37.7)	114(62.3)	1.32(.61, 2.86)	5.33(1.63, 17.40)**
Others	9(29.0)	22(71.0)	.89(.31, 2.56)	2.95(.67, 13.09)
**Education of women**				
No formal education	9(11.8)	67(88.2)	1	1
Primary school (1–8)	52(37.4)	87(62.6)	4.45(2.05, 9.67)	5.78(1.97, 17.02)**
Secondary and above	73(55.7)	58(44.3)	9.37(4.31, 20.37)	16.69(4.62, 60.31)**
**Occupation of women**				
Gov't/NGO employee	27(54.0)	23(46.0)	1	1
Merchant	49(59.0)	34(41.0)	1.23(.61, 2.49)	1.40(.45, 4.38)
Housewife	51(27.0)	138(73.0)	.32(.17, .60)	.27(.09, .79)**
Daily laborer	7(29.2)	17(70.8)	.35(.12, .99)	.14(.02, .80)**
**Occupation of husbands**				
Gov't/NGO employee	74(49.3)	76(50.7)	1	1
Merchant	36(33.0)	73(67.0)	.51(.30, .85)	.47(.14, 1.56)
Daily laborer	7(14.6)	41(85.4)	.18(.07, .42)	.20(.04, 1.11)
Other	17(43.6)	22(56.4)	.79(.39, 1.61)	.65(.13, 3.31)
**Household income**				
<1500	59(30.4)	135(69.6)	1	1
1500–3000	29(46.8)	33(53.2)	2.01(1.12, 3.61)	2.22(.70, 7.06)
>3000	46(51.1)	44(48.9)	2.39(1.43, 4.00)	1.17(.37, 3.73)
**Radio in the household**				
Yes	66(35.3)	121(64.7)	1	1
No	68(42.8)	91(57.2)	1.37(.89, 2.12)	1.52(.77, 3.01)
**Have television in the HH**				
Yes	101(41.1)	145(58.9)	1	1
No	33(33.0)	67(67.0)	.71(.43, 1.15)	.40(.16, .99)**
**Parity**				
Nulliparous	5(35.7)	9(64.3)	1.10(.35, 3.47)	.54(.08, 3.45)
1–2	28(35.9)	50(64.1)	1.12(.62, 2.00)	.50(.19, 1.32)
3–4	56(46.7)	64(53.3)	1.73(1.04, 2.87)	1.52(.67, 3.45)
5+	45(33.6)	89(66.4)	1	1
**History of abortion**				
Yes	14(15.6)	76(84.4)	.21(.11, .39)	7.17(2.81, 18.27)**
No	120(46.9)	136(53.1)	1	1
**Decision maker on fertility**				
Wife	13(23.6)	42(76.4)	.58(.35, .96)	.12(.04, .37)**
Husband	33(33.0)	67(67.0)	.36(.18, .72)	.77(.34, 1.78)
Jointly	88(46.1)	103(53.9)	1	1
**Knowledge about RLTC**				
Good	49(26.9)	133(73.1)	.34(.22, .54)	1.02(.46, 2.27)
Poor	85(51.8)	79(48.2)	1	1
**Attitude about RLTC**				
Good	40(26.0)	114(74.0)	.37(.23, .58)	2.85(1.46, 5.57)**
Poor	94(49.0)	98(51.0)	1	1

## Discussion

This study documented that about 38.7% of married women in the reproductive age group use RLTCs. We observed higher prevalence in the current study as compared to similar studies in Ethiopia, Indonesia and elsewhere (5,6,11–14). This difference might be due to the sociodemographic variability in the study areas, all of our study participants were from urban areas and might have more access to family planning information. A comparable prevalence was reported from Eastern Ethiopia(15).

Protestant Christian religion followers had much higher odds of using RLTCs as compared to their Muslim counterparts. This finding is supported by a study conducted in Nigeria, where the use of contraceptives was significantly lower among Muslim women as compared to women from other religions (16). This might be due to the belief that contraceptive use contradicts with Islam teaching because Islam encourages Muslims to give birth to many children(17,18). Another possible reason is that polygamy is permitted in Islam and most of the women in polygamous marriage believe that they can win the attention of their husbands when they often get pregnant for them (19).

In consistence with studies from different parts of Ethiopia and Zambia, RLTC utilization increased with advanced educational status (6,20–22). This might be because advanced educational status increased the aware of women on the benefits of RLTCs. The decision making power is also higher for women with a better education. A different finding was reported from Indonesia, where no significant association was reported between educational status and RLTCs utilization (14). This difference might be due to the small sample size used in the above study.

The utilization of RLTCs was lower for housewives and daily laborers in comparison to government or NGO employees. This finding is consistent with other studies from Ethiopia where government employees were more likely to utilize RLTCs (12,23). The possible explanation might be that government and NGO employees have a better educational status and in turn better access to information.

In consistence with studies in Ethiopia and Central Asia, the utilization of RLTCs was lower for those who do not have radios in the household compared to their counterparts (12,24). The possible reason might be that listening to family planning messages through electronic media had improved the chance of using RLTCs (25).

We observed that women with history of abortion had higher odds of using RLTCs. Similarly, a study from Angola reported that women with history of abortion were more likely to use modern family planning compared to women who never had abortion (26). The possible reasons might be access for LTRCs and health education given at health facilities as part of post-abortion care (PAC).

Compared to women who decide jointly with their partners, women who decide alone on fertility had lower odds of using RLTCs. This was in agreement with studies in Ethiopia, Zambia, and Uganda, where women who discuss with husbands were more likely associated with RLTCs utilization (11,12,27–31).Women with a better attitude towards RLTCs had much higher odds of using RLTCs than their counterparts. This finding is consistent with other studies from Ethiopia (27, 32, 33).

We used cross-sectional data and a cause-effect relationship could not be measured. To conclude, women should be empowered educationally through other alternative opportunities to school, for the the reason that it would or would not be possible to bring all married women to school. In addition to electronic media, different community events and community conversations should be used to convey messages on contraceptives particularly RLTCs since it is the most cited source of information on family planning in Ethiopia (10). Programs working on contraceptives utilization should also encourage spousal communication. Behavioral change communications would benefit women in shaping their attitudes towards RLTCs.
